# Acculturation, Education, and Cognition in Older Asian Americans and Canadians: results from the Asian Cohort for Alzheimer's Disease study

**DOI:** 10.1002/alz70860_106670

**Published:** 2025-12-23

**Authors:** Yian Gu, Dolly Reyes‐Dumeyer, Pei‐Chuan Ho, Wai Haung Yu, Jennifer S. Yokoyama, Badri N. Vardarajan, Gyungah R Jun, Donghe Li, Guerry M. Peavy, Boon Lead Tee, Haeok Lee, Tiffany W Chow, Victor W. Henderson, Van Ta Park, Richard Mayeux, Li‐San Wang, The Asian Cohort for Alzheimer's Disease (ACAD)

**Affiliations:** ^1^ Departments of Neurology and Epidemiology, the Sergievsky Center, Columbia University Irving Medical Center, New York, NY, USA; ^2^ Taub Institute for Research on Alzheimer's Disease and the Aging Brain, Columbia University, New York, NY, USA; ^3^ The Gertrude H. Sergievsky Center, College of Physicians and Surgeons, Columbia University, New York, NY, USA; ^4^ G. H. Sergievsky Center, Vagelos College of Physicians and Surgeons, Columbia University, New York, NY, USA; ^5^ Department of Neurology, Vagelos College of Physicians and Surgeons, Columbia University, and the New York Presbyterian Hospital, New York, NY, USA; ^6^ The Leonard and Davis Institute of Health Economics, University of Pennsylvania, Philadelphia, PA, USA; ^7^ University of Toronto, Toronto, ON, Canada; ^8^ Memory and Aging Center, Department of Neurology, Weill Institute for Neurosciences, University of California, San Francisco, San Francisco, CA, USA; ^9^ The Taub Institute for Research on Alzheimer's Disease and the Aging Brain, Vagelos College of Physicians & Surgeons, Columbia University, New York, NY, USA; ^10^ Department of Neurology, Columbia University, New York, NY, USA; ^11^ Biomedical Genetics Section, Department of Medicine, Boston University Chobanian & Avedisian School of Medicine, Boston, MA, USA; ^12^ Boston University Chobanian & Avedisian School of Medicine, Boston, MA, USA; ^13^ Department of Neurosciences, University of California San Diego, San Diego, CA, USA; ^14^ Memory and Aging Center, Department of Neurology, University of California San Francisco, San Francisco, CA, USA; ^15^ Meyers College of Nursing, New York University, New York, NY, USA; ^16^ Penn Neurodegeneration Genomics Center, Department of Pathology and Laboratory Medicine, University of Pennsylvania Perelman School of Medicine, Philadelphia, PA, USA; ^17^ Stanford University, Stanford, CA, USA; ^18^ School of Nursing, Department of Community Health Systems, University of California, San Francisco, CA, USA; ^19^ Department of Epidemiology, Mailman School of Public Health, Columbia University, New York, NY, USA; ^20^ Department of Neurology, Taub Institute for Research on Alzheimer's Disease and the Aging Brain, Columbia University, New York, NY, USA

## Abstract

**Background:**

Acculturation—adapting to a new culture—plays a significant role in shaping health outcomes among immigrants, including older Asian Americans and Canadians (ASACs). Education, a well‐established protective factor for cognition, may interact with acculturation to influence cognitive health. This study examines the relationship between acculturation and cognition among ASACs and explores how acculturation interacts with education.

**Method:**

This cross‐sectional study included 622 dementia‐free ASAC participants from the Asian Cohort for Alzheimer's Disease (ACAD). An acculturation score (ranging 1‐7, with higher score indicating more acculturation) was constructed based on the sum of nativity level (place of birth and years in the U.S. or Canada, ranging 1‐4) and English proficiency (ranging 1‐3), self‐reported in English, Chinese, Korean, and Vietnamese. Participants were categorized into three acculturation groups: separation (score 1‐3, preference for Asian culture), integration (4‐5, balanced preference), and assimilation (6‐7, well adoption of new culture). Cognition was assessed using the Cognitive Abilities Screening Instrument (CASI) and Common Objects Memory Test (COMT). Multivariable linear regressions were used to examine the associations between acculturation and cognition, adjusted for age, gender, education, and study site. The interaction between acculturation and education on cognition was also evaluated.

**Result:**

Participants were 71.7 years (SD=7.1) old, 68.3% were women, and most were first‐generation immigrants (97%) raised in Asia (94%). About 34%, 41% and 25% were in the separation, integration, and assimilation groups, respectively. Each one‐unit increase in acculturation was associated with a 0.39‐point higher CASI score (SE=0.20, *p* = 0.042), but no association was observed with COMT. A significant negative interaction (*p* <0.001 for CASI, *p* <0.01 for COMT) was found between education and acculturation: the positive association of education with cognition was weaker in the assimilation and integration groups compared to the separation group.

**Conclusion:**

Higher levels of acculturation may compensate for the impact of low education on cognition in older ASACs. The findings suggest considering both education and acculturation in understanding cognitive health among aging immigrant populations. Future research incorporating socioeconomics and other contextual factors is needed to better understand the relationship among acculturation, education, and cognitive health.

